# In Situ Photoacoustic Detection System for SO_2_ in High-Pressure SF_6_ Buffer Gas Using UV LED

**DOI:** 10.3390/s22249846

**Published:** 2022-12-14

**Authors:** Wei Hu, Kang Li, Tunan Chen, Zongjia Qiu, Guoqiang Zhang

**Affiliations:** 1 State Key Laboratory of Power Grid Environmental Protection, China Electric Power Research Institute, Wuhan 430074, China; 2 Institute of Electrical Engineering, Chinese Academy of Sciences, Beijing 100190, China; 3 University of Chinese Academy of Sciences, Beijing 100190, China

**Keywords:** SO_2_ gas detection, UV LED, photoacoustic spectroscopy, high-pressure buffer gas, pressure correction

## Abstract

Sulfur dioxide (SO_2_) is a key indicator for fault diagnosis in sulfur hexafluoride (SF_6_) gas-insulated equipment. In this work, an in situ photoacoustic detection system using an ultraviolet (UV) LED light as the excitation source was established to detect SO_2_ in high-pressure SF_6_ buffer gas. The selection of the SO_2_ absorption band is discussed in detail in the UV spectral regions. Based on the result of the spectrum selection, a UV LED with a nominal wavelength of 285 nm and a bandwidth of 13 nm was selected. A photoacoustic cell, as well as a high-pressure sealed gas vessel containing it, were designed to match the output optical beam and to generate a PA signal in the high-pressure SF6 buffer gas. The performance of the proposed system was assessed in terms of linearity and detection limit. An SO_2_ detection limit (1σ) of 0.17 ppm was achieved. Additionally, a correction method was supplied to solve PA signal derivation induced by pressure fluctuation. The method can reduce the derivation from about 5% to 1% in the confirmation experiment.

## 1. Introduction

Because of its excellent insulation and arc extinction abilities, sulfur hexafluoride (SF_6_) has been widely used in power equipment such as GIS (gas-insulated switchgear), GIL (gas-insulated transmission line), GIT (gas-insulated transformer), gas insulated bushing, and so on, since the 1960s [1,2]. With the development and long-term operation of SF_6_-insulated equipment, the insulation status monitor for this sort of equipment has long been a hot topic for researchers. Several monitoring techniques have been proposed, such as ultra-high frequency (UHF) [3], ultrasonic method [4], and so on. However, these detection techniques are vulnerable to electromagnetic interference or noise vibration. Additionally, these techniques can only be used to detect discharge faults. Therefore, it is necessary to find a non-invasive method that is invulnerable to electromagnetic interference to properly evaluate the insulation state of SF_6_-insulated equipment online.

Though SF_6_ is a stable and non-toxic gas, it will decompose when there are overheating or discharging faults and generate a series of sulfides. Some of the sulfides will react with water or oxygen in equipment to generate SO_2_, H_2_S, SO_2_F_2_, and so on [5,6,7]. These decomposition gases can be poisonous and corrosive but useful. Plenty of researchers at home and abroad have verified that the internal insulation state can be estimated based on these gases [8,9,10]. The generation of decomposition products indicates an insulation fault: overheating or discharging or both. Among all of the decomposition gases, SO_2_ is the most important for fault identification [11]. Therefore, an online detection technology for SO_2_ is widely sought.

Several techniques have been proposed for trace SO_2_ detection including gas chromatography, the chemical sensor method, infrared and UV absorption spectroscopy [12,13], infrared and UV photoacoustic spectroscopy [14,15,16], etc. Among those methods, gas chromatography requires a carrier gas, is expensive, and is a method normally used for laboratory testing or patrol inspections. Meanwhile, the sample gas is contaminated. Chemical methods can also only achieve offline detection due to the limited sensor lifetime when immersed in an SO_2_ mixture. Due to the existence of crossover interference and low infrared absorption coefficient, it is hard to obtain high detection sensitivity for SO_2_ in small volumes using an infrared absorption band. Some researchers [16] proposed UV absorption spectroscopy using a UV electrode-less lamp as a light source to detect SO_2_ in SF_6_, and this method reached a detection limit (DL) of 0.67 ppm. However, the high volume and weight of this device make it unsuitable for online detection. Moreover, all of these methods mentioned are designed for the detection of SO_2_ at normal pressures and cannot be used in a high-pressure SF_6_ environment directly. Currently, there are two ways to use these machines to measure SO_2_. One way is to reduce the gas pressure, sample, test and finally to discharge the sample gas; the other way is to reduce the gas pressure; to sample, test, and raise the pressure; and to recharge the sample gas. For the first way, the sample gas is consumed and is not suitable for online monitoring; the second way needs a special gas circuit of which the complexity, risk of leakage, and price are high, which increases the difficulties in application.

In this paper, an in situ photoacoustic (PA) detection system for SO_2_ in a high-pressure SF_6_ buffer gas using UV LED is presented. The detection theory of SO_2_ using UV LED photoacoustic spectroscopy (PAS) is outlined briefly. Then, the absorption characteristics of SO_2_ in the UV range are studied and a well-matched UV LED light is selected to obtain good detection system performance. Next, a gas vessel, as well as a PA cell for high-pressure gas detection, is designed. Finally, the characteristics of the proposed detection system are tested using a standard SO_2_ gas mixture. The new UV source, a novel gas vessel, and PA cell design resulted in an SO_2_ detection device that can be used to directly monitor SO_2_ concentrations in high-pressure SF_6_ gas.

## 2. Detection Theory of SO_2_ PA Detection System

**Figure 1:**
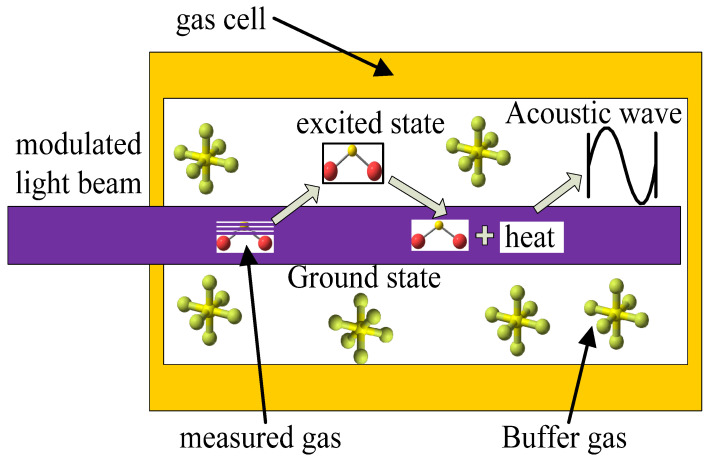
Schematic diagram of PAS.

The theoretical basis of the gas PA effect is schematically shown in Figure 1. The SO_2_ PA detection system is based on the gas PA effect.

The modulated light beam enters the gas cell and transmits along the axis of the gas cell. The gas molecules absorb the light at certain wavelengths and are excited from the ground state to the excited state. Some of the excited gas molecules transition back to the ground state via nonradiative transition and release the absorbed energy in the form of molecular average kinetic energy. The internal energy of the gas molecules ascends. Consequently, acoustic waves are generated by this periodic heating. These acoustic waves can be measured using a microphone and are converted into electrical signals.

Generally, the PA signal of a cylindrical cavity non-resonant PA cell can be expressed by the following equation [17]:

(1) $$S_{PA}=s_{m}P_{input}F_{cell}\alpha c$$

where *S_PA_* is the PA signal, *s_m_* is the sensitivity of the microphone, *P_input_* is the power of the light source emitted into the PA cell, *F_cell_* is the PA cell constant, *α* is the absorption coefficient, the product of *P_input_* and *α* is the effective absorption power, and *c* is concentration of the SO_2_ gas.

From Equation (1), the PA signal is proportional to sensitivity, PA cell constant, and effective absorption power. Therefore, three ways to increase the PA signal exist. One is to select a microphone with higher sensitivity; the second is to design a PA cell with a bigger PA constant; and the third is to select a proper light source and absorption band to obtain a larger effective absorption power.

It can also be seen from Equation (1) that for a settled PA system, the PA signal *S_PA_* is proportional to the gas concentration *c*. Therefore, once the relationship between those two variables is calibrated, the concentration *c* of an unknown gas mixture can be calculated by the measured PA signal directly. Meanwhile, it should be noted that the calibration curve is related to the test conditions such as pressure and temperature. The absorption coefficient, PA cell constant, and sensitivity of the used microphone are all related to pressure and temperature. 

In this paper, we only consider the effect of pressure. The absorption coefficient can be expressed as follows:

(2) $$\alpha =\sigma N=\sigma \cdot \frac{P}{P_{0}}\cdot N_{0}=\alpha_{0}\cdot \frac{P}{P_{0}}$$

where *σ* is absorption cross-section of the target gas and is usually a constant if the pressure varies in a small range, *α*_0_ is the absorption coefficient at the reference pressure, *N* is the molecule number per unit volume, *P* is the gas pressure, and *N*_0_ is the Loschmidt constant at the reference pressure *P*_0_.

**Figure 2:**
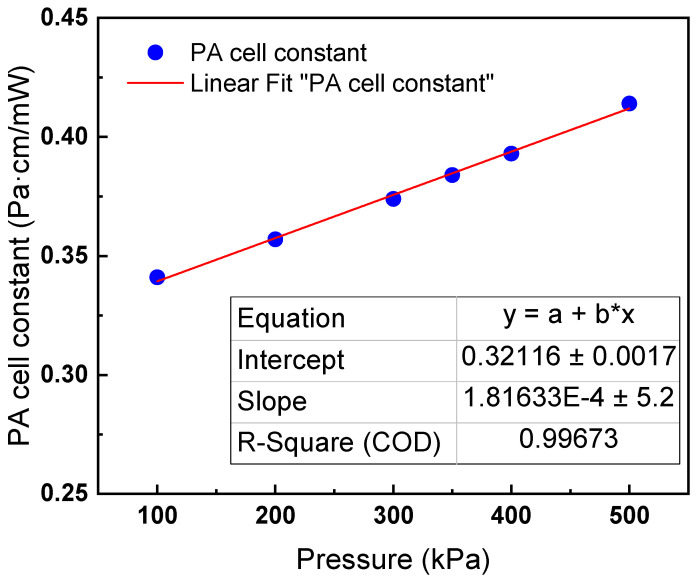
The PA cell constant along with gas pressure.

Then, the PA cell constant of a cylindrical cavity non-resonant PA cell can be expressed as follows:

(3) $$F_{cell}=\frac{i(\gamma -1)}{\omega \pi a^{2}[1+(i/\omega \tau_{0})]}$$

(4) $$\tau_{0}\approx \rho C_{P}a^{2}/(2.5\kappa )$$

where γ is specific heat ratio of the buffer gas, *ω* is modulated frequency of the source light, *a* is the cross-sectional radius of the PA cell, *κ* is thermal conductivity of the buffer gas, *ρ* is the gas density of the buffer gas, and *C_P_* is heat capacity at a constant pressure of the buffer gas. The parameters and calculated PA cell constant (*a* = 6 mm, *ω* = 2*π* × 40) at 30 °C are listed in Table 1. Additionally, the PA cell constant along with pressure is plotted in Figure 2. Figure 2 shows that the relationship between them is approximately linear when the pressure increases from 100 kPa to 500 kPa. The PA cell constant can be approximately expressed as

(5) $$F_{cell}=a\times P+b$$

The influence of static pressure on the sensitivity of a microphone can be expressed as

(6) $$s_{m}=s_{m0}\cdot \frac{100}{(100-F)+F\cdot P/P_{ref}}$$

where *s_m_*_0_ is the sensitivity of the microphone under reference static pressure *P_ref_*, *P* is the static pressure, and *F* is the fraction of air stiffness in percent at a reference static pressure (ratio between air stiffness and total diaphragm system stiffness) and is dependent on the microphone design, typically 0.1. The relative sensitivity decreases from 1 to 0.996 as gas pressure increases from 100 kPa to 500 kPa. The effect of the microphone on the PA signal is relatively small.

Based on the above analysis, Equation (1) can be changed to

(7) $$\begin{array}{l} S_{PA}=s_{m0}\cdot \frac{100}{(100-F)+F\cdot P/P_{ref}}\cdot P_{input}\cdot (a\cdot P+b)\cdot P\cdot \alpha_{0}c\\ \;=s_{m0}P_{input}\alpha_{0}c\cdot f(P) \end{array}$$

**Figure 3:**
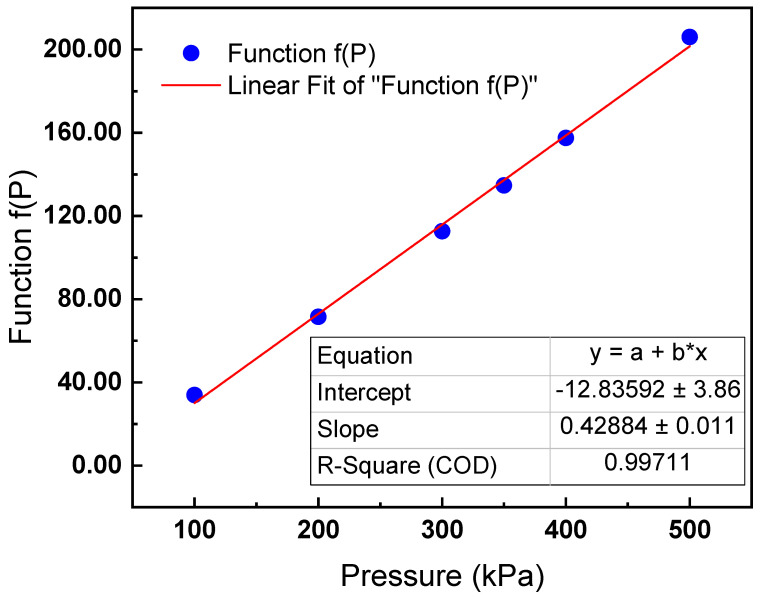
Function *f(P)* changes along with gas pressure.

Function *f(P)* can be calculated using the abovementioned parameters. The results are shown in Figure 3.

Based on the above analysis, the relationship between the PA signal and the gas pressure can be approximately linear when the pressure change is small.

## 3. Establishment of the Detection System

### 3.1. Selection of SO_2_ Detection Wavelength and Excitation Light Source

**Figure 4:**
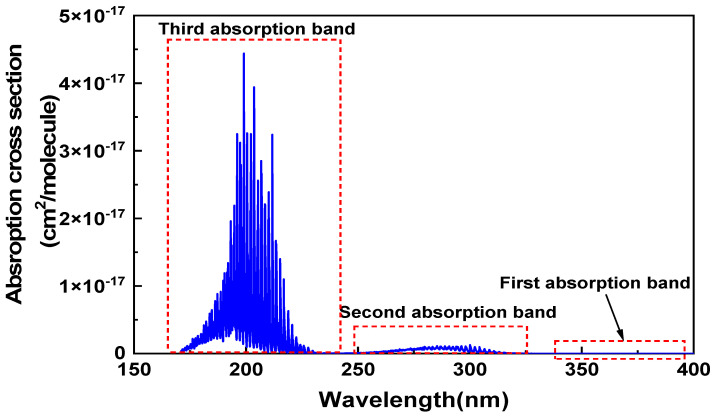
Absorption characteristics of SO_2_ in the range from 180 nm to 400 nm.

According to previous research results [18,19], SO_2_ has three absorption bands in its UV absorption spectrum, as shown in Figure 4. The first one is 340–400 nm, with the strongest absorption at 370 nm, but it is a very weak absorption region. The second is 240–330 nm, which is a strong absorption region. The third one is 180–240 nm, which is a very strong absorption region. However, UV light with a wavelength below 240 nm will cause dissociation of SO_2_ and is not suitable for PA detection. The second absorption band is selected as a potential spectrum segment.

**Figure 5:**
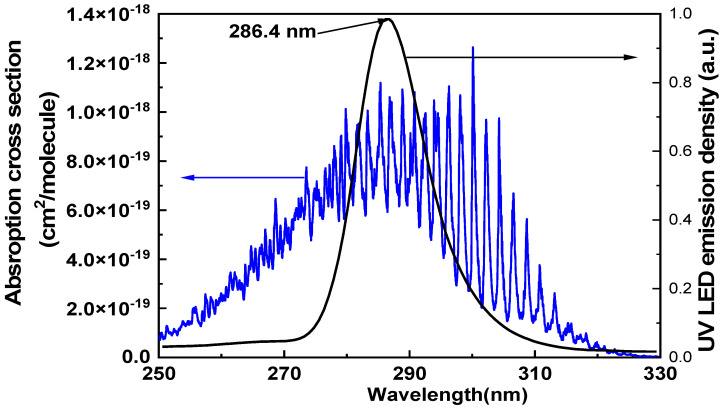
The emission spectrum of LED and absorption characteristics of SO_2_ in the range from 250 nm to 330 nm.

To improve the detection capability, crossover interference should be avoided as much as possible. Luckily, there is no or very weak absorption among 240–330 nm for all the SF_6_ decomposition gases. To reach the chosen range, a UV LED (M285L5) with a nominal wavelength of 285 nm and a bandwidth of 13 nm was selected as the light source. The typical output power of the LED can reach 70 mW. The emission spectrum of the UV LED is demonstrated in Figure 5. The absorption cross sections of SO_2_ from 250 nm to 310 nm are depicted in Figure 5 as well. It can be seen the actual central wavelength of the LED is 286.4 nm and that the bandwidth is about 12.5 nm. Meanwhile, the emission spectrum of the LED can cover most of the absorption band of SO_2_. This coverage makes strong excitement of the PA signal possible. Moreover, a condenser lens (aspheric lens UV-Coated, ASL2520-UV) is used to decrease affections of the divergence angle of the LED source and to increase the PA effect.

### 3.2. Gas Vessel Design for High-Pressure Gas Detection

**Figure 6:**
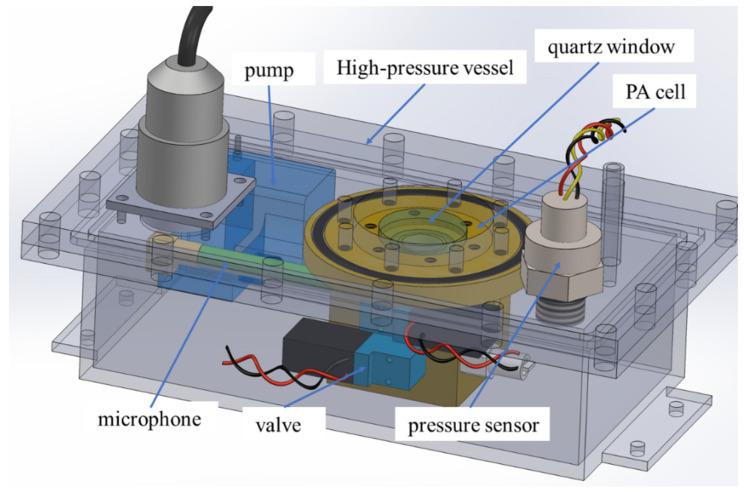
Three-dimensional model of the designed gas vessel and PA cell.

To realize the measurement of high-pressure gas, a gas vessel is specially designed, as shown in Figure 6. The PA cell, microphone, solenoid valves, and other components are all fixed into the high-pressure gas vessel, and the UV-light beam enters the gas chamber of the PA cell through a quartz window. The pressure sensor is fixed on cover plate of the vessel. The main reasons to use a double-layer structure are as follows: The increase in gas pressure increases the sealing difficulty of the microphone; moreover, the pressure tolerance ability of micro solenoid valves and the microphone is weak, and the risk of gas leakage increases when the pressure rises.

The PA cell is the core component of the detection device, and its performance directly affects the detection properties. The structure of the non-resonant PA cell used in this paper is shown in Figure 6. The gas chamber is a cylindrical structure. The inner diameter of the gas chamber is 12 mm, and the length is 35 mm. The gas inlet and outlet were designed to be in the side wall of the cylindrical cavity and are sealed using the valve. The light inlet window is made of quartz glass with good UV light transmission properties and high mechanical strength, as the window is used for light transmission and gas seal. The thickness of the quartz window is estimated according to the following formula [20].

(8) $$\delta_{g}=\sqrt{\frac{3p_{c}A(3m+1)}{8\pi m\sigma_{\max}}}$$

where *δ_g_* is calculated thickness of the quartz window; *p_c_* is the calculated pressure and depends on the design working pressure, considering 1.5 times the safety factor, 0.7 MPa here; *A* is the area of the quartz window; *m* is the reciprocal of Poisson’s ratio and, for quartz glass, is 5.882; and *σ_max_* is ultimate tensile strength of the quartz window and, for quartz glass, it is 73.45.

### 3.3. Basic Structure of Detection System

**Figure 7:**
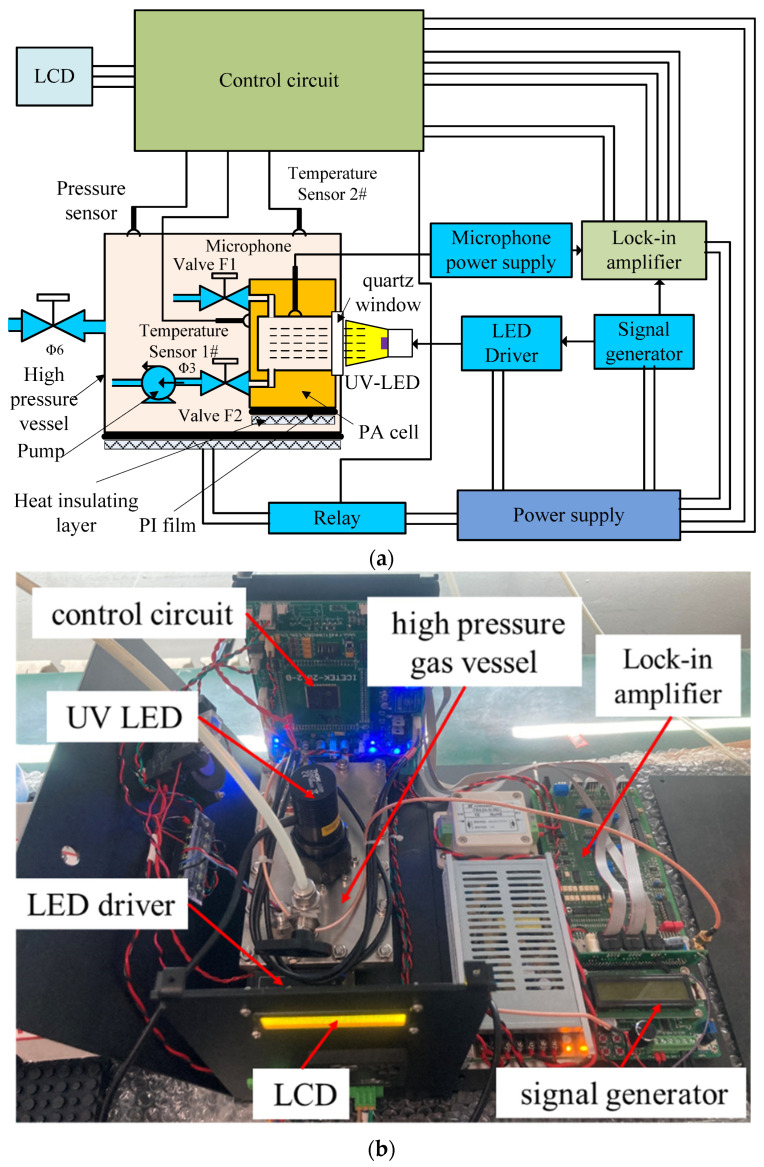
UV LED-based SO_2_ detection system: (**a**) schematic diagram and (**b**) practicality picture.

Based on the UV PA theory and the analysis above, a detection system for SO_2_ was established, as shown schematically in Figure 7.

From Figure 7, a signal generator was employed to generate the LED driver (LEDD1B) modulation signal as well as the lock-in amplifier (LIA-BVD-150-L, FEMTO, Germany) reference signal. The LED driver modulation signal was a sine waveform with DC bias, the amplitude of the wave was set as 5 V, and the frequency can be altered. The LED driver was set to Modulation Mode. The output current of the LED driver could follow the amplitude of the input sine wave. The LED driver was connected to the selected UV LED. At the same time, a TTL synchronization signal was output to the lock-in amplifier by the signal generator. The PA signal was collected by the microphone and transferred to the lock-in amplifier. The amplified signal was sampled and processed by the control circuit board. Then, the results were displayed on the LCD. Sensors were used to monitor temperature of the PA cell and gas pressure of the high-pressure vessel. The temperature of the PA cell was controlled using a PI heating film.

## 4. Test Results and Analysis of Detection System

### 4.1. Quantitative Detection and Performance Assessment

The temperature of the PA cell was controlled at 30 °C, the gas pressure inside the high-pressure gas vessel and PA cell was controlled at 500 kPa, and the modulation frequency of UV LED was set at 40 Hz. The lock-in amplifier works in 1f mode as the UV LED is a broad-spectrum light. Standard SO_2_ gas (bought from Beijing Haipubeifen gas product company) with concentrations of 8.6 ppm, 71.5 ppm, 127.5 ppm, 161.3 ppm, and 248.1 ppm was tested. Before each gas intake, the gas vessel and PA cell were evacuated less than 1 kPa. Then, standard SO_2_ with certain concentrations was filled into the high-pressure gas vessel and PA cell slowly to avoid pressure shock to the microphone. After the detection system warmed up, 100 successive data points were collected at a sampling frequency of 20 Hz, and the average amplitude was calculated. After one measurement at a certain concentration of SO_2_ was completed, the evacuating procedure was repeated to clean the SO_2_ mixture gas in the gas cell to avoid affecting the next detection. Then, another standard SO_2_ gas was filled into the vessel and PA cell for measurement.

**Figure 8:**
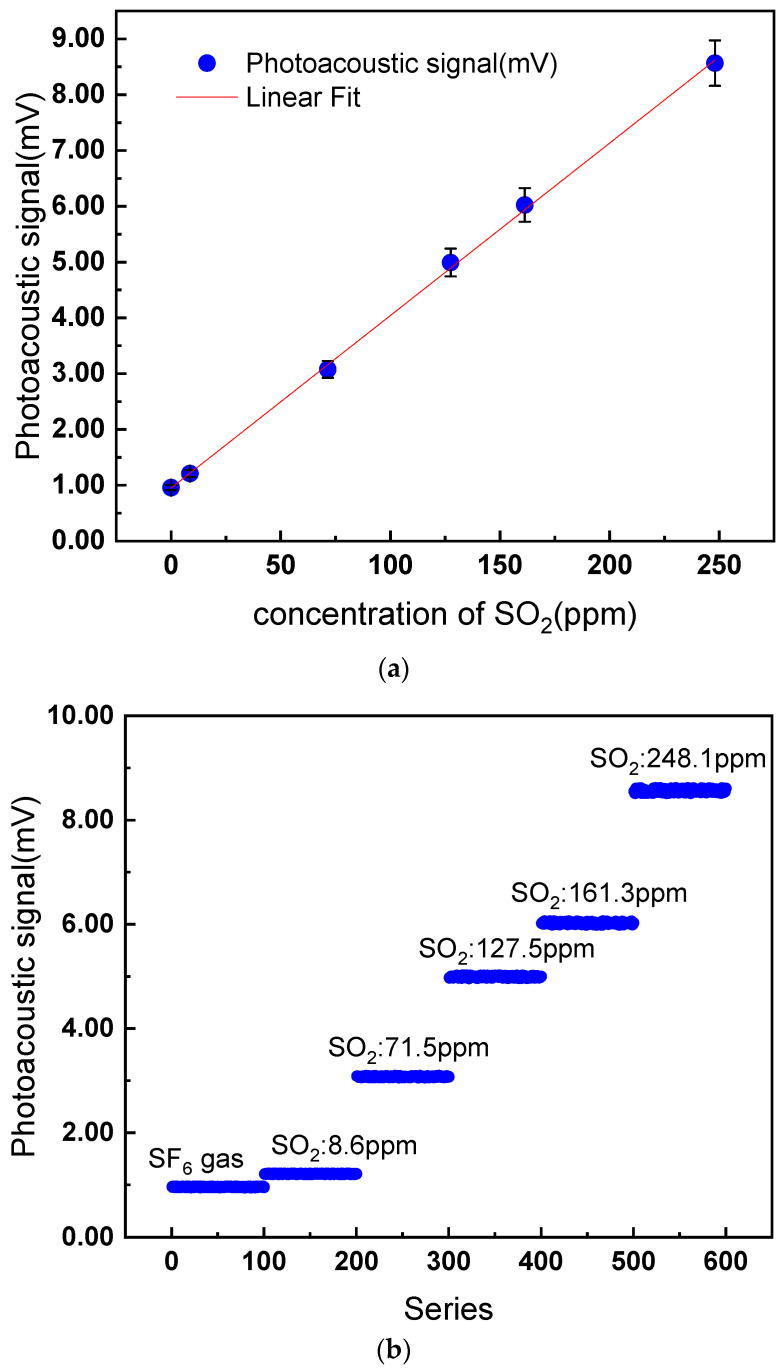
Detection properties of the SO_2_ detection system: (**a**) the linearity of the PA signal response and (**b**) series of measured results of different SO_2_ concentrations.

Based on the data points, the relationship between the corresponding PA signals and the concentrations of SO_2_ is depicted in Figure 8.

From Figure 8, the linear fitting R^2^ value > 0.998 confirmed the linearity of the PA signal response to the concentration of SO_2_. Additionally, the linear fitting function, also called calibration curve, can be expressed as follow at 30 °C and 500 kPa:

(9) $$\begin{array}{c} S_{PAS}=h(c)=0.0309c+0.963\\ \mathrm{or}\;c=g(S_{PAS})=32.30S_{PAS}-30.85 \end{array}$$

where *S_PAS_* is the PA signal and *c* is the gas concentration. The unknown concentration of the SO_2_ gas mixture can be calculated using Equation (9) once the corresponding PA signal was obtained.

To obtain the repeatability of the proposed detection system, the measurements were repeated six times. The measured SO_2_ gas concentration was 248.1 ppm, the gas pressure was 500 kPa, and the temperature was controlled at 30 °C. For each test, a new gas mixture was used to simulate the real application. The measured PA signal and gas concentration are listed in Table 2. The results of the experiment indicated that the relative standard deviation of PA signal and gas concentration were both within 1%, much better than the actual requirements, which proved the feasibility of the detection system presented in this paper.

Normalized noise equivalent absorption (NNEA) [21,22] and detection limit (DL) [23] are used to indicate the property of PAS. NNEA is usually used for PA sensors using a laser source. Therefore, DL was employed to estimate the capability of our detection system. The DL *c*_min_ is expressed as

(10) $$c_{\min}=k\sigma_{b}/\delta_{s}$$

where *k* is the risk coefficient; *σ_b_* is fluctuation of the background, that is, the standard deviation of the background signal, which is 5.24 µV by measuring pure SF_6_ gases; and *σ_d_* is the fluctuation of the detection signal. It can be considered that the background fluctuation is equal to the signal fluctuation at a trace level, namely *σ_b_* = *σ_d_*. *δ_s_* is sensitivity. It is usually indicated by the response value corresponding to the measured object per unit and is the slope of Equation (9), which is 30.9 µV/ppm. Therefore, the DL (k = 1) of the detection system can be determined by the experimental data and is 0.17 ppm, which can meet the requirement of industrial applications.

**Figure 9:**
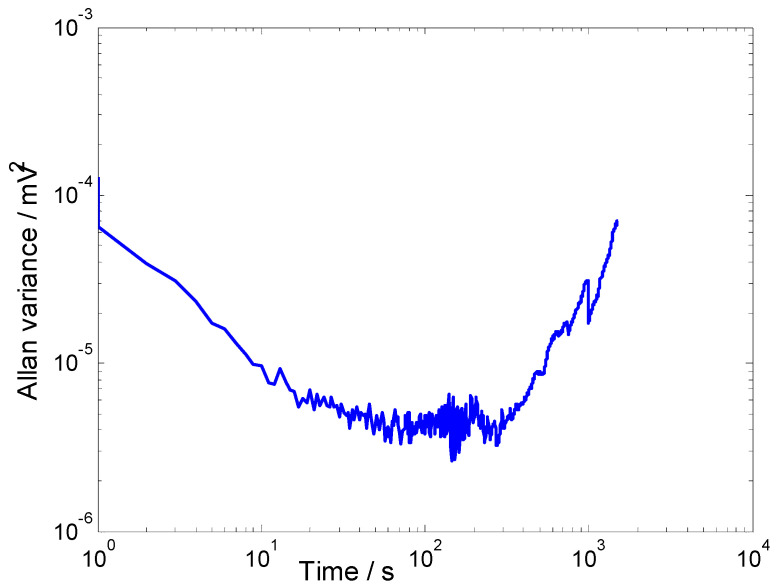
Allan variance analysis of the detection system.

To evaluate the stability of the detection system, the PA cell was filled with pure SF_6_ gas, and then, an experimental test lasting 3000 s was performed. The Allan variance curve is shown in Figure 9. The minimum Allan variance is ~2.63 × 10^−6^ mV^2^. Moreover, when evaluating the minimum DL of a measuring instrument, the root means square of the Allan variance can usually be used as the minimum standard deviation of the measuring instrument. In this work, the minimum standard deviation is $\sqrt{2.63\times 10^{-6}{\;\mathrm{mV}}^{2}}=\sim 1.62\;\mu V$. Therefore, according to Equation (10), the minimum DL of the system is ~52 ppb.

**Figure 10:**
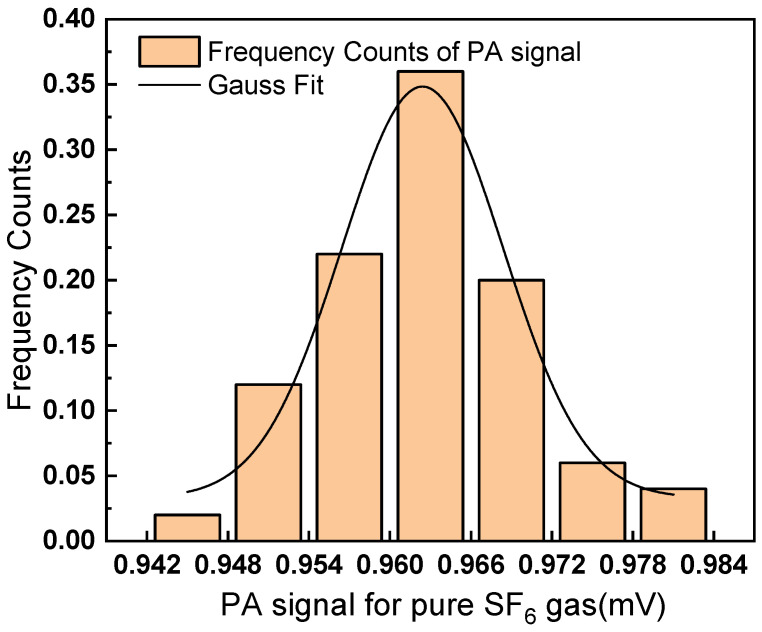
Frequency of the 50 times test results for pure SF_6_ gas.

To perform the measurement precision analysis, the pure SF6 gas was measured 50 times continuously. The measured results were plotted in a column graph, as shown in Figure 10. The horizontal axis represents the PA signal range, and the vertical axis represents the frequency count. Then, the graph was fitted with a Gaussian profile. The expected value of the Gaussian distribution is 0.962 mV, the standard deviation is 5.98 µV, and the half-width at half maximum (HWHM) is 7.05 µV.

### 4.2. Pressure Properties and Correction Method

**Figure 11:**
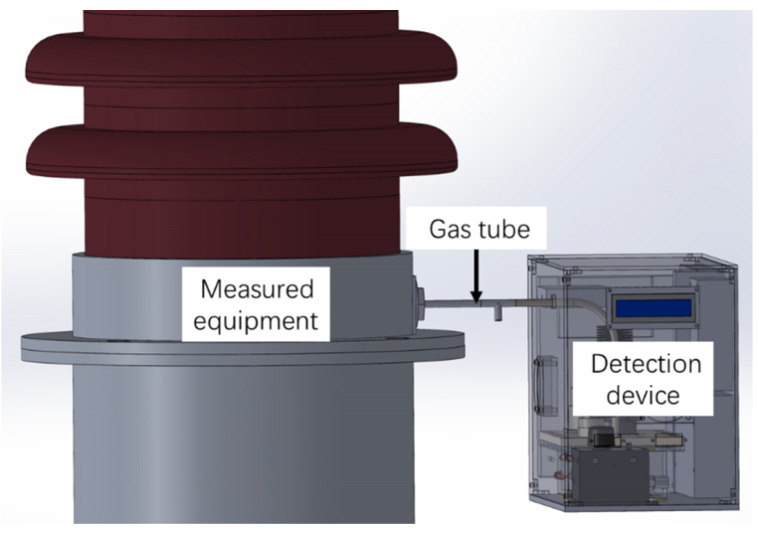
Schematic diagram of the connection between measured equipment and the proposed SO_2_ detection device.

In field applications, the detection device is usually connected to GIS or GIT through a gas tube, as shown in Figure 11. The gas needs to be exchanged between GIS and the detection device directly to make sure the detected components are gases from GIS, not dead gases in the detection device. However, the gas pressure and temperature in GIS or GIT will fluctuate with operating conditions such as ambient temperature and load status. The operating ambient temperature of GIS may fluctuate from −40 °C to 45 °C, and the pressure may fluctuate from 350 kPa to 550 kPa or higher. This means that the gas pressure and temperature will also fluctuate in the detection device. The temperature in the device can be controlled relatively easily by the heating and cooling module, while the control of gas pressure is relatively difficult. Therefore, the pressure properties and correction method for the proposed SO_2_ measuring device are studied in this part.

#### 4.2.1. Pressure Properties of the Detection System

**Figure 12:**
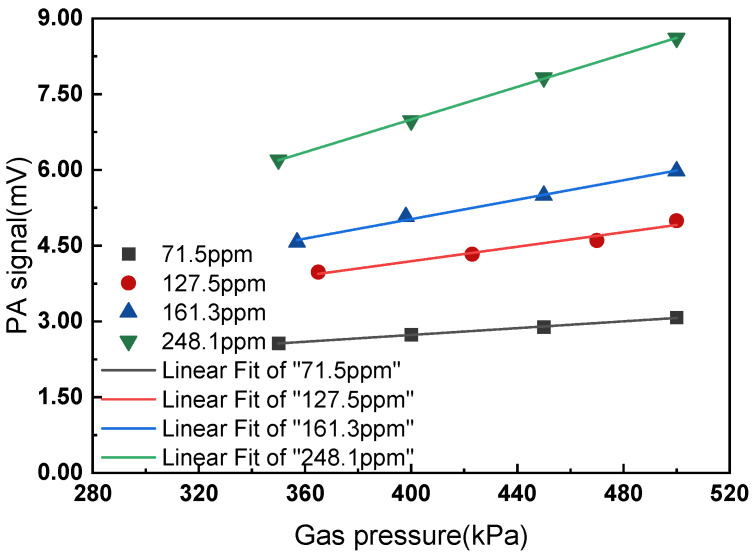
Linear fitting of gas pressure and PA signal.

Temperature of the PA cell was controlled at 30 °C, and the modulation frequency of the UV LED was controlled at 40 Hz. Standard SO_2_ gases with concentrations of 71.5 ppm, 127.5 ppm, 161.3 ppm, and 248.1 ppm were tested using the mentioned method. The pressure of the gas mixture varies from about 350 to 500 kPa. The measurement results are shown in Figure 12. The linear fitting parameters and deviation induced by pressure fluctuation are listed in Table 3.

From Figure 12 and Table 3, we can see the PA signal increase nearly linearly as gas pressure increases and is consistent with theoretical analysis. Meanwhile, the growth rate of the PA signal is different under different gas concentrations. Two conclusions can be obtained. First, the gas pressure has a great influence on the PA signal amplitude and gas detection results, which needs to be corrected; second, the gas pressure has different effects on the PA signal at different gas concentrations, which means there is not a constant correction factor to correct the calibration curve.

#### 4.2.2. Correction Method for Pressure Fluctuation

**Figure 13:**
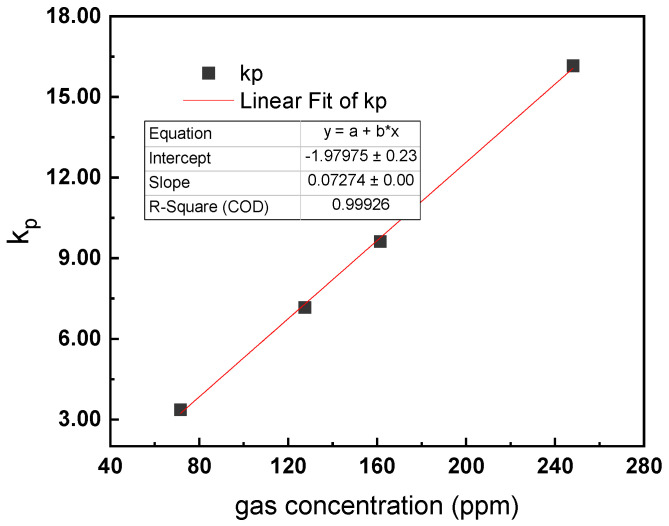
Linearity property of *k_p_* vs. gas concentration c.

According to the experimental data in Figure 12 and Table 3, when the gas volume fraction *c* is fixed, the PA signal of the mixture gas increases gradually with the increase in the gas pressure, and the relationship is approximately linear. The slope of the change can be recorded as *k_p_*. When the gas volume fraction *c* changes, *k_p_* will also change. The relationship between *k_p_* and the gas volume fraction c can be obtained by fitting the experimental data, as shown in Figure 13.

As shown in Figure 13, R-square is 0.999. The relationship is also approximately linear and can be expressed as follows:

(11) $$k_{p}=f(c)=a_{1}\times c+b_{1}$$

where *a*_1_ is 0.0727 and *b*_1_ is −1.980 for the presented system.

The variation in the gas PA signal caused by the change in gas pressure can be represented by the following model:

(12) $$\Delta S_{P}=S_{P}-S_{0}=kp\cdot (P-P0)=f(c)\cdot (P-P0)$$

where *S_P_* is the measured PA signal, *S*_0_ is the PA signal corrected from *S_P_* to reference pressure, *P*_0_ is the reference pressure, and *P* is the measured gas pressure.

**Figure 14:**
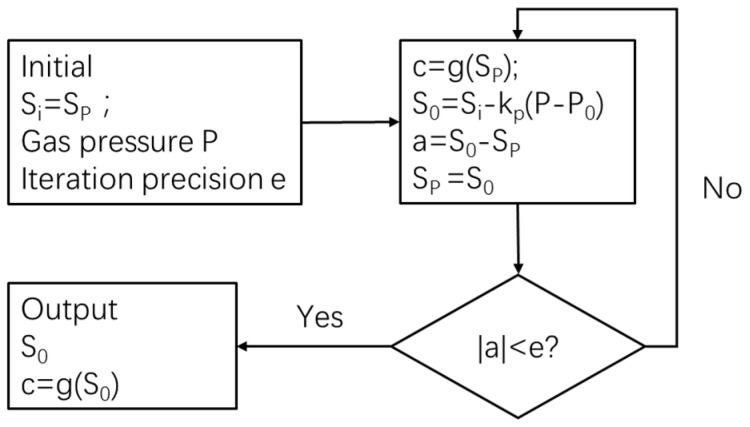
Flowchart of the correction iteration process.

The essence of the pressure correction model is to correct the measured PA signal *S_P_* to the PA signal *S*_0_ under the reference pressure and then to calculate the actual gas concentration through Equation (9). It can be seen from Equation (12) that the gas volume fraction is needed to calculate Δ*S_P_*, but the gas volume fraction is unknown, so it is necessary to use an iterative method to obtain the corrected result. The iteration process is shown in Figure 14.

To verify the credibility of the above iterative model, three concentrations of standard gases, 68.7 ppm, 137.4 ppm, and 274.4 ppm, at 30 °C under two different pressures were measured. Then, the measured PA signal was adjusted using the correction model. The iterative accuracy was not greater than 10^−4^. The gas concentrations were calculated based on the measured signals before and after correction. The results are listed in Table 4. The results indicate that the correction model can effectively reduce the detection deviation caused by gas pressure fluctuation.

## 5. Conclusions

In this paper, an in situ photoacoustic detection system of SO_2_ gas in high-pressure SF_6_ buffer gas was developed using UV LED. Based on PAS theory, the effective absorption power, which is the product of the absorption cross-section and light power, is a key factor determining the PA signal. Accordingly, a UV LED well-matched with a strong absorption band of SO_2_ was selected as the excitation source. A high-pressure vessel and a matched PA cell were designed. Then, a PA detection system of SO_2_, which can directly connect to measuring equipment by gas tube, was established. The detection characteristic of the system was estimated. The DL (*k* = 1) of the presented detection system was achieved at 0.17 ppm when the gas pressure was up to 500 kPa. Meanwhile, a pressure correction method was supplied to solve the problem of pressure fluctuation. Experimental results indicate that gas concentration derivation due to pressure fluctuation can decrease from about 5% to 1%. The results in this paper illustrate that the developed instrument can meet the requirement of SO_2_ detection in the monitoring of SF_6_ gas-insulated equipment.

## Figures and Tables

**Table 1:** Parameters and calculated PA cell constant (*a* = 6 mm, *ω* = 2*π* × 40).

Pressure (kPa)	Density (kg/m^3^)	*Cp* (kJ/kg-K)	*Cp/Cv*	Therm.Cond. (mW/m-K)	PA Cell Constant (Pa·cm/mW)
100	5.8566	0.67673	1.0968	13.365	0.341
200	11.842	0.68109	1.1014	13.410	0.357
300	17.964	0.68575	1.1064	13.458	0.374
350	21.079	0.68820	1.1091	13.484	0.384
400	24.231	0.69073	1.1118	13.511	0.393
500	30.652	0.69607	1.1176	13.568	0.414

**Table 2:** Repeatability of the detection system.

Serial No	PA Signal (mV)	SO_2_ Concentration (ppm)
1	8.748	251.7
2	8.650	248.6
3	8.610	247.3
4	8.635	248.1
5	8.615	247.4
6	8.612	247.3
Average value	8.645	248.4
Repeatability (%)	0.558	0.627

**Table 3:** Linear fitting parameters and measured deviation induced by pressure fluctuation.

SO_2_ Gas Concentration (ppm)	Linear Fitting Parameters			Gas Pressure Fluctuation 40 kPa	
	Slope (µV/kPa)	Intercept (mV)	R^2^ Value	PA Signal Deviation (µV)	Gas Concentration Deviation (ppm)
71.5	3.37	1.385	0.998	134.8	4.4
127.5	7.17	1.324	0.953	286.8	9.2
161.3	9.62	1.176	0.991	384.8	12.4
248.1	16.17	0.530	0.999	646.8	20.9

**Table 4:** Effect of pressure fluctuation correction model.

Standard Gas Concentration (ppm)	Gas Pressure (kPa)	Measured Gas Concentration Before Correction	Error Before (%)	Measured Gas Concentration after Correction	Error After (%)
68.7	470	65.2	−5.09	68.1	−0.87
	520	71.9	4.66	69.9	1.75
137.4	470	129.1	−6.04	136.9	−0.36
	520	143.6	4.51	138.4	0.73
274.7	470	255.9	−6.84	373.3	−0.51
	520	288.3	4.95	276.6	0.69

## Data Availability

Data sharing is not applicable.
